# Identification of an Important Orphan Histidine Kinase for the Initiation of Sporulation and Enterotoxin Production by *Clostridium perfringens* Type F Strain SM101

**DOI:** 10.1128/mBio.02674-18

**Published:** 2019-01-22

**Authors:** John C. Freedman, Jihong Li, Eric Mi, Bruce A. McClane

**Affiliations:** a Department of Microbiology and Molecular Genetics, University of Pittsburgh School of Medicine, Pittsburgh, Pennsylvania, USA; University of Oklahoma Health Sciences Center; Tufts University School of Medicine; Monash University

**Keywords:** *Clostridium perfringens*, enterotoxin, histidine kinase, sporulation

## Abstract

Clostridium perfringens type F strains cause a common human foodborne illness and many cases of nonfoodborne human gastrointestinal diseases. Sporulation plays two critical roles during type F enteric disease. First, it produces broadly resistant spores that facilitate type F strain survival in the food and nosocomial environments. Second, production of C. perfringens enterotoxin (CPE), the toxin responsible for causing the enteric symptoms of type F diseases, is restricted to cells in the process of sporulation. While later steps in the regulation of C. perfringens sporulation have been discerned, the process leading to phosphorylation of Spo0A, the master early regulator of sporulation and consequent CPE production, has remained unknown. Using an insertional mutagenesis approach, the current study identified the orphan histidine kinase CPR0195 as an important factor regulating C. perfringens sporulation and CPE production. Specifically, a CPR0195 null mutant of type F strain SM101 made 10^3^-fold fewer spores than its wild-type parent and produced no detectable CPE. In contrast, a null mutant of another putative C. perfringens orphan histidine kinase (CPR1055) did not significantly affect sporulation or CPE production. Studies using a *spoIIA* operon promoter-driven reporter plasmid indicated that CPR0195 functions early during sporulation, i.e., prior to production of sporulation-associated sigma factors. Furthermore, *in vitro* studies showed that the CPR0195 kinase domain can autophosphorylate and phosphorylate Spo0A. These results support the idea of CPR0195 as an important kinase that initiates C. perfringens sporulation by directly phosphorylating Spo0A. This kinase could represent a novel therapeutic target to block C. perfringens sporulation and CPE production during type F disease.

## Abstract (precis)

Clostridium perfringens type F enteric diseases, which include a very common form of food poisoning and many cases of antibiotic-associated diarrhea, develop when type F strains sporulate and produce C. perfringens enterotoxin (CPE) in the intestines. Spores are also important for transmission of type F disease. Despite the importance of sporulation for type F disease and the evidence that C. perfringens sporulation begins with phosphorylation of the Spo0A transcriptional regulator, the kinase phosphorylating Spo0A to initiate sporulation and CPE production had not been ascertained. In response, the current report now provides identification of an orphan histidine kinase named CPR0195 that can directly phosphorylate Spo0A. Results using a CPR0195 null mutant indicate that this kinase is very important for initiating C. perfringens sporulation and CPE production. Therefore, the CPR0195 kinase represents a potential target to block type F disease by interfering with intestinal C. perfringens sporulation and CPE production.

## INTRODUCTION

The clostridia include several important pathogens of humans and domesticated animals. An important feature of those pathogenic clostridial species is their ability to form spores that contribute to environmental persistence and disease transmission. Examples of diseases where clostridial spores contribute to transmission include *Clostridium* (*Clostridioides*) *difficile* infection, foodborne or infant botulism, tetanus, and Clostridium perfringens clostridial myonecrosis (gas gangrene).

Similarly, spores also play a significant role in the transmission of several important human enteric diseases caused by C. perfringens. Among the foremost examples is C. perfringens type F food poisoning, formerly known as one of the forms of type A food poisoning prior to the recent expansion of the C. perfringens isolate typing scheme (1). Type F food poisoning, the second most common bacterial foodborne illness in the United States, is caused by type F (formerly type A) strains producing C. perfringens enterotoxin (CPE) (2). Most type F food poisoning strains make spores exhibiting exceptional resistance to food environment stresses such as those resulting from exposure to heat, cold, and food preservatives (3, 4). Those extreme spore resistance properties are largely attributable to the type F food poisoning strains producing a variant of small acid soluble protein 4 (SASP-4) which binds more tightly to spore DNA than the SASP-4 made by most other C. perfringens strains (5, 6). This tight DNA binding by their SASP-4 variant offers spores of type F food poisoning strains exceptional protection against stresses such as heat stress, thus facilitating survival of these strains in temperature-abused foods. Those spores later germinate into vegetative cells, which then rapidly multiply in foods. Enteric disease develops after the contaminated food is consumed. Spores are also important for the transmission of CPE-associated nonfoodborne diseases (NFD) caused by type F strains. Type F NFDs, which include about 5% to 10% of all antibiotic-associated diarrhea cases, are thought to be transmitted by ingestion of spores, often from the nosocomial environment (7, 8).

Sporulation also contributes to another critical aspect of type F strain pathogenicity. Production of CPE, which is necessary for the enteric virulence of all type F strains, is sporulation dependent (9–11). During type F enteric diseases, C. perfringens sporulates in the intestines and produces CPE (2). The enterotoxin accumulates in the cytoplasm of the mother cell until it is released into the intestinal lumen when the mother cell lyses to free the endospore. CPE then binds to receptors on enterocytes, forms a pore, and induces intestinal damage (10).

In both clostridial and *Bacillus* spp., the process of sporulation involves a precisely regulated cascade of gene expression (12–14). Like other clostridia and *Bacillus* spp., C. perfringens sporulation-related gene expression is largely regulated by 4 alternative sigma factors named sigma E (SigE), SigF, SigG, and SigK (15, 16). Western blotting studies using sigma factor null mutants indicated that in C. perfringens, during sporulation, SigF production is necessary to make SigE, SigG, and SigK (16). An additional study using *sigE* and *sigF* mutants demonstrated that SigE and SigK directly control expression of the *cpe* gene during sporulation (15).

In Bacillus subtilis, the paradigm species for bacterial sporulation studies, the onset of sporulation involves a phosphorelay pathway that is amplified considerably when KinA, one of several kinases comprising the relay, autophosphorylates upon sensing appropriate environmental cues (12, 13, 17). This phosphorelay climaxes with the phosphorylation of Spo0A, the master transcriptional regulator of sporulation. Phosphorylated Spo0A then increases the expression of early sporulation genes, including those encoding early sporulation-related sigma factors.

Interestingly, the phosphorelay pathway for initiating sporulation in B. subtilis is not present in the clostridia (12, 13). Instead, the nonpathogenic species Clostridium acetobutylicum and Clostridium thermocellum initiate their sporulation using orphan histidine kinases that lack a cognate response regulator (18, 19). For the pathogenic clostridia, some evidence suggests that orphan kinases also play a role in initiating sporulation (20, 21), although this is less established (see Discussion) despite the importance of sporulation for clostridial pathogenesis.

More specifically, while it has been demonstrated that Spo0A is required for C. perfringens sporulation and CPE production (22), no kinase(s) has yet been identified that phosphorylates Spo0A to initiate sporulation and to signal the onset of CPE production by type F strains. Seven putative orphan histidine kinases are annotated in the genome of type F strain SM101 (23) and might be involved in initiating sporulation and CPE production. Therefore, the current study used a TargeTron-mediated insertional mutagenesis approach to inactivate genes encoding two of those putative kinases. Phenotypic testing of those null mutants revealed that one of the two genes encodes a protein that is critically important for the induction of sporulation and CPE production. This protein was shown to act upstream of the operon encoding SigF (an early sigma factor required for C. perfringens to produce the other sporulation-associated sigma factor genes [16]), and *in vitro* biochemical analyses confirmed that this protein is a kinase that can autophosphorylate and then directly phosphorylate Spo0A. Taking the results together, this study identified an orphan histidine kinase that plays a major role in the early induction of C. perfringens sporulation and CPE production.

## RESULTS

### Bioinformatic identification of putative orphan kinases in C. perfringens type F strain SM101.

The SM101 genome (23) contains seven putative orphan kinase genes, annotated as *cpr0195*, *cpr1055*, *cpr1316*, *cpr1493*, *cpr1728*, *cpr1953*, and *cpr1954*, that might be involved in initiating sporulation and CPE production. With two exceptions, these putative orphan kinase genes are also present in 7 other surveyed GenBank C. perfringens genomes (strain 13, F4969, ATCC 13124, ATCC 3626, JGS1495, JGS1721, and JGS1987), representing all C. perfringens types except type G. The two exceptions both involve type C strain JGS1495, which does not carry the *cpr1055* gene or the *cpr1316* gene. Six of the putative orphan kinase genes are predicted by the PSORTb program (https://psort.org/psortb/) to encode histidine kinases with membrane localization, although the prediction is less clear for the seventh gene; i.e., c*pr1055* is predicted to encode a kinase with two transmembrane domains, but its predicted structure does not match that of a typical membrane-associated histidine kinase. Only limited (<60%) amino acid sequence similarity exists among these seven putative histidine kinases, with this limited similarity residing within the predicted kinase domain of these proteins. These putative kinases also do not share significant homology with KinA/KinB/KinC of B. subtilis or with histidine kinases of other clostridial species.

### Construction and characterization of a SM101 *cpr0195* null mutant and complementing strain.

To determine which, if any, of the putative orphan histidine kinase genes play an important role in initiating sporulation and CPE production in C. perfringens strain SM101, attempts were initiated to inactivate the putative orphan kinase genes by insertional mutagenesis using a *Clostridium*-modified TargeTron group II intron.

**FIG 1:**
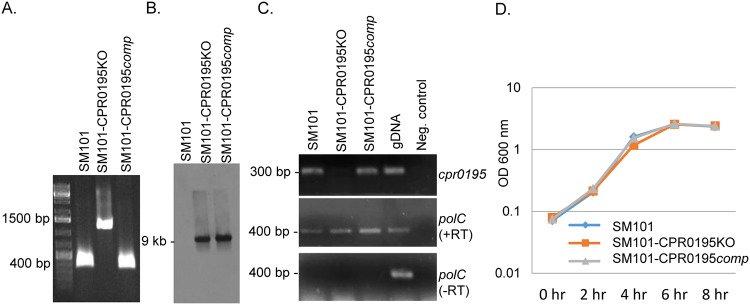
Characterization of the SM101-CPR0195KO null mutant and SM101-CPR0195comp complementing strain. (A) PCR confirming insertional mutagenesis of the *cpr0195* gene in SM101-0195KO. Shown is the *cpr0195* PCR product ampliﬁed using DNA from wild-type SM101 (lane 2), the SM101-CPR0195KO mutant (lane 3), or the SM101-CPR0195comp complementing strain (lane 4). Note that, compared to the ∼300-bp product amplified using DNA containing a wild-type *cpr0195* gene, DNA from the null mutant strain supported amplification of a larger (∼1,200-bp) product due to the insertion of an intron into its *cpr0195* gene. (B) Southern blot hybridization of an intron-specific probe with DNA from SM101 (left), SM101-CPR0195KO (middle), or SM101-CPR0195comp (right). DNA from each strain was digested overnight with EcoRI at 37°C and then electrophoresed on a 1% agarose gel. The size of the hybridizing band in the middle and right lanes is shown to the left. Using DNA from wild-type SM101, no intron-speciﬁc band was detected, while a single intron-speciﬁc band was detected for the SM101-CPR0195KO mutant and complementing strain. (C) RT-PCR analysis for *cpr019*5 (top panel) or *polC* (middle panel) transcription in wild-type SM101, the SM101-CPR0195KO mutant, or the complementing strain. SM101 DNA was used as a positive control (gDNA [genomic DNA]). PCRs lacking template DNA acted as a negative control. To show that the RNA preparations from the three strains were free from DNA contamination, these samples were also subjected to PCR without reverse transcription (bottom panel). (D) Growth curves for wild-type SM101, the SM101-CPR0195KO mutant, and the SM101-CPR0195comp strain cultured at 37°C in MDS medium for up to 8 h. Aliquots of each culture were measured every 2 h for their OD_600_. All experiments were repeated three times, and mean representative values are shown. The markers used in panels A and C were Thermo Fisher 1-kb DNA ladders.

A group II intron was successfully inserted into the *cpr0195* gene, as verified by PCR (Fig. 1A). Using primers corresponding to internal *cpr0195* open reading frame (ORF) sequences that are adjacent to the intron insertion site, PCR amplification resulted in an ∼300-bp product from wild-type SM101. However, using the same primers, an ∼1,200-bp product was amplified from the putative *cpr0195* null mutant, which is consistent with insertion of the ∼900-bp intron into the *cpr0195* ORF. A Southern blotting experiment performed using an intron-specific probe (Fig. 1B) confirmed the presence of a single intron insertion in this putative mutant, which was named SM101-CPR0195KO (where “KO” represents knockout). Reverse transcription-PCR (RT-PCR) demonstrated that this mutant had lost expression of the *cpr0195* gene (Fig. 1C). Growth curve analyses showed that this mutant grew similarly to wild-type SM101 in modified Duncan-Strong (MDS) sporulation medium (Fig. 1D).

A complementing strain was prepared by ligating the wild-type *cpr0195* gene (including 800 bp of upstream sequence) into the pJIR750 shuttle plasmid (24) and then electroporating the resultant plasmid into SM101-CPR0195KO to create SM101-CPR0195comp. PCR amplified a 300 bp product using internal *cpr0195* primers, demonstrating the presence of a wild-type copy of the *cpr0195* gene in this complementing strain (Fig. 1A). Southern blot analyses showed (Fig. 1B) that the complementing strain still retained the intron insertion, as expected. Although Fig. 1B demonstrated the presence of the disrupted *cpr0195* gene in this strain, it was not amplified in the experiment represented by Fig. 1A because the primers used would amplify products from both the wild-type and intron-disrupted *cpr0195* genes in this strain but the much smaller PCR product amplified from the wild-type gene was created more rapidly and thus was greatly increased in relative abundance after each PCR round. This phenomenon has been observed previously (5, 25, 26). Importantly, RT-PCR confirmed that the complementing strain had regained expression of the *cpr0195* gene (Fig. 1C). The complementing strain grew similarly to wild-type SM101 or SM101-CPR0195KO in MDS medium (Fig. 1D).

### CPR0195 is a strong positive regulator of SM101 sporulation and CPE production.

Using wild-type SM101, the SM101-CPR0195KO mutant, and the SM101-CPR0195comp complementing strain, an experiment was performed to assess whether the CPR0195 putative orphan histidine kinase is involved in regulating C. perfringens sporulation and CPE production. After each strain was grown for 16 h in MDS medium at 37°C to allow spore formation, the cultures were subjected to heat shock treatment for 20 min at 70°C to kill vegetative cells and to promote germination of any heat-resistant spores present in the culture and were then plated to enumerate spores by colony counting.

**FIG 2:**
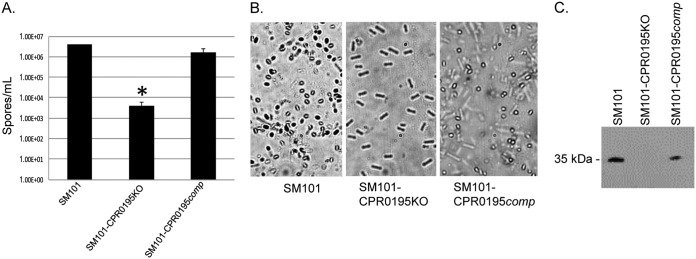
CPR0195 controls sporulation and CPE production in C. perfringens strain SM101. (A) Wild-type (WT) SM101, SM101-CPR0195KO, and SM101-CPR0195comp were grown overnight at 37°C in MDS, and the cultures were then subjected to heat shock treatment and plated on BHI agar. After overnight incubation in an anaerobic jar, the resultant colonies were counted and the counts were converted to numbers of spores per milliliter. Mean results from three repetitions are shown, and error bars represent the standard errors. The asterisk (*) indicates that the *cpr0195* mutant formed significantly (*P* < 0.05) fewer spores than the wild-type or complementing strains. (B) Photomicroscopy of WT SM101, SM101-CPR0195KO, and SM101-CPR0195comp grown in MDS, confirming the plate count results. (C) Supernatants of WT SM101, SM101-CPR0195KO, and SM101-CPR0195comp grown overnight at 37°C in MDS were assessed by Western blotting for CPE, showing that detectable CPE production is abolished by knockout of the *cpr0195* gene. The blot shown is representative of results of three repetitions.

Importantly, that analysis revealed that inactivating the *cpr0195* gene caused a statistically significant (*P* < 0.03), >1,000-fold decrease in the number of spores present in MDS cultures (Fig. 2A). This decrease was attributable to mutation of the *cpr0195* gene, rather than to a secondary mutation, since complementation of the mutant to restore expression of the *cpr0195* gene also significantly increased sporulation (Fig. 2A). Photomicroscopy confirmed the presence of abundant spores in MDS cultures of the wild-type and complementing strains but not in MDS cultures of the SM101-CPR0195KO mutant (Fig. 2B).

Supernatants from the same cultures used for Fig. 2A experiments were also subjected to SDS-PAGE and Western blotting using a rabbit polyclonal antiserum raised against purified CPE to assess the presence of CPE. Relative to wild-type SM101 cultures, cultures of the SM101-CPR0195KO mutant showed an absence of CPE production. However, CPE production was restored by complementation with a plasmid carrying the wild-type *cpr0195* gene (Fig. 2C). Taken together, these results indicate that the *cpr0195*-encoded CPR0195 protein is a very important positive regulator of both sporulation and CPE production in C. perfringens.

### Construction of a *cpr1055* null mutant.

**FIG 3:**
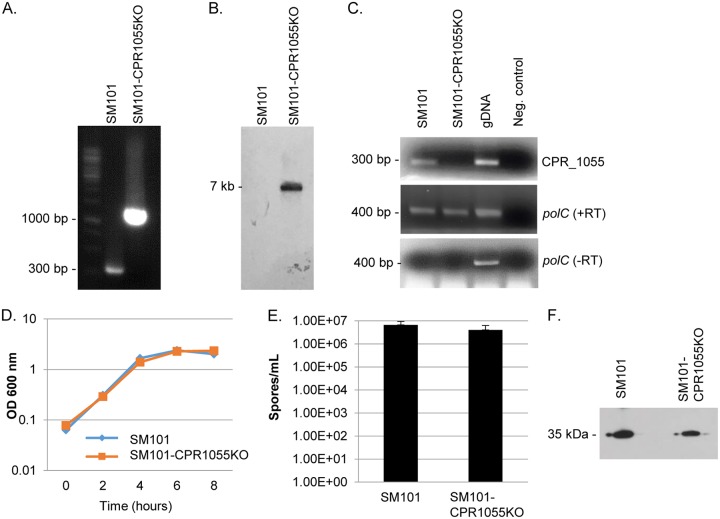
Characterization of the SM101-CPR1055KO null mutant and analysis of sporulation and CPE production. (A) PCR confirming insertional mutagenesis of th*e cpr1055* gene in SM101-CPR1055. Shown is the *cpr1055* PCR product ampliﬁed using DNA from wild-type SM101 (left lane) or the SM101-CPR1055KO mutant (right lane). Note that DNA from the null mutant strain supported amplification of a larger product due to the insertion of an intron into its *cpr1055* gene. (B) Southern blot hybridization with an intron-specific probe with DNA from SM101 or SM101-CPR1055KO. The blot shows results of intron-speciﬁc Southern blot hybridization with DNA from wild-type SM101 (left lane) or the *cpr1055* null mutant (middle lane). DNA from each strain was digested overnight with EcoRI at 37°C and then electrophoresed on a 1% agarose gel. The size of the hybridizing band in the right lane is shown to the left. Using DNA from wild-type SM101, no intron-speciﬁc band was detected. However, a single intron-speciﬁc band was detected for the SM101-CPR1055KO mutant. (C) RT-PCR analysis for *cpr1055* (top panel) or *polC* (middle panel) transcription in wild-type SM101 or the SM101-CPR1055KO mutant. SM101 DNA was used as a positive control (gDNA). PCRs lacking template DNA acted as a negative control. To show that the RNA preparations from both strains were free from DNA contamination, the samples were also subjected to PCR without reverse transcription (bottom panel). (D) Growth curves for wild-type SM101 versus the SM101-CPR1055KO mutant cultured at 37°C in MDS medium for up to 8 h. Aliquots of each culture were measured every 2 h for their OD_600_. (E) Comparison of results of sporulation by WT SM101 versus SM101-CPR1055KO. Both strains were grown overnight at 37°C in MDS and then subjected to heat shock treatment and plated on BHI agar. After overnight incubation in an anaerobic jar, the resultant colonies were counted and the counts were converted to numbers of spores per milliliter. (F) Comparison of levels of CPE production by SM101 versus the SM101-CPR1055KO mutant. Supernatants of WT SM101 or SM101-CPR1055KO were grown overnight at 37°C in MDS and then assessed by Western blotting for CPE. The results showed that CPE production remained strong after inactivation of the *cpr1055* gene. All experiments were repeated three times, and mean representative values are shown. The markers used in panels A and C were Thermo Fisher 1-kb DNA ladders.

To distinguish whether (i) there was specificity with respect to the strong effects of CPR0195 on sporulation and CPE production that were observed in Fig. 2 experiments or (ii) all C. perfringens orphan histidine kinases had similarly strong impacts on sporulation and CPE production, attempts were continued to construct additional knockouts of genes encoding other putative orphan histidine kinases in SM101. This effort proved successful in generating a CPR1055 null mutant, which was named SM101-CPR1055KO. The identity of this mutant was confirmed by PCR using primers spanning the *cpr1055* intron insertion site. This analysis demonstrated an ∼900-bp increase in the PCR product size amplified using DNA from SM101-CPR1055KO versus wild-type SM101 (Fig. 3A). Southern blotting using a probe specific for the group II intron revealed that only a single intron had inserted into the genome of SM101-CPR1055KO (Fig. 3B). The presence of this intron insertion in the mutant eliminated *cpr1055* expression, as shown by reverse transcriptase PCR (Fig. 3C). SM101-CPR1055KO exhibited growth rates similar to those of wild-type SM101 in MDS medium (Fig. 3D).

### A functional *cpr1055* gene is not required to induce substantial sporulation and CPE production.

The availability of the *cpr1055* mutant allowed us to test whether inactivation of any one putative orphan kinase gene of SM101 would be sufficient to sharply reduce the initiation of sporulation and CPE production. Comparing the levels of CPE production and sporulation in the wild-type and SM101-CPR1055KO strains, no significant (*P* = >0.4) reduction in sporulation was observed for the SM101-CPR1055KO mutant relative to wild-type SM101 (Fig. 3E). Similarly, CPE Western blotting showed that this *cpr1055* mutant continued to strongly produce CPE (Fig. 3F). Because the mutant does not show a significant phenotype, no complementing strain was prepared or characterized.

### Expression kinetics of the *cpr0195* and *cpr1055* genes.

**FIG 4:**
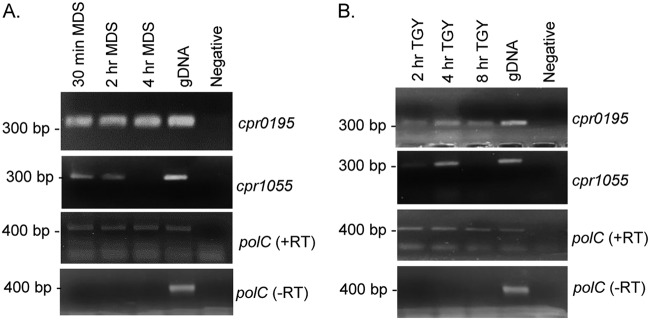
Expression analysis of the *cpr0195* and *cpr1055* genes for SM101 grown under vegetative and sporulation conditions. SM101 was grown at 37°C in MDS sporulation medium (A) or TGY vegetative growth medium (B) for the indicated times. Cell pellets were collected by centrifugation, RNA was harvested, and expression at each time point under each condition was assessed. Results representative of three repetitions are shown.

In order for SM101 to initiate sporulation upon sensing an environmental signal, the gene encoding an orphan histidine kinase involved in initiating sporulation should be expressed under vegetative growth conditions such that the kinase should already be available to phosphorylate Spo0A when the vegetative cell receives the appropriate signal to commence sporulation. Therefore, reverse transcriptase PCR was used to assess expression of the *cpr0195* gene by SM101 in early sporulating cultures (Fig. 4A) and vegetative cultures (Fig. 4B). For comparison, expression of the *cpr1055* gene was similarly evaluated in sporulating and vegetative cultures of this strain.

Consistent with the CPR0195 protein being already available when the sporulation signal arrives, RT-PCR showed that the *cpr0195* gene was expressed under all culture conditions assayed, which included all growth phases in vegetative media or MDS (not shown). In contrast, expression of the *cpr1055* gene turned off after 4 h of growth in MDS and during late-logarithmic-phase vegetative culture growth.

### The CPR0195 protein affects early steps in sporulation prior to expression of genes encoding the sigma factor cascade.

To determine whether the CPR0195 protein affects sporulation during early or late stages of sporulation, a previously prepared *spoIIA* operon promoter-GusA fusion reporter plasmid was used (27). The rationale for this experiment was based on results from our previous Western blot study (16), which indicated that SigF (encoded by the *spoIIA* operon) is produced early during sporulation since its production is necessary for C. perfringens to make detectable amounts of the other three sporulation-associated sigma factors. Since a putative Spo0A binding site is present upstream of the *spoIIA* operon in C. perfringens (this study) and since *spo0A* is essential for sporulation of this bacterium (22), Spo0A-regulated expression of the *spoIIA* operon encoding SigF should occur early during C. perfringens sporulation.

Wild-type SM101, the SM101-CPR0195KO mutant, and the SM101-CPR1055KO mutant carrying the reporter plasmid were each grown overnight in MDS medium. Note that the SM101-CPR0195comp strain could not be used in these studies because of apparent issues of plasmid incompatibility between the *spoIIA* reporter plasmid and the complementation plasmid (not shown). Following growth of those strains, the beta-glucuronidase substrate 6 mM 4-nitrophenyl-β-d-glucuronide was added to the supernatants and levels of GusA activity were then determined.

**FIG 5:**
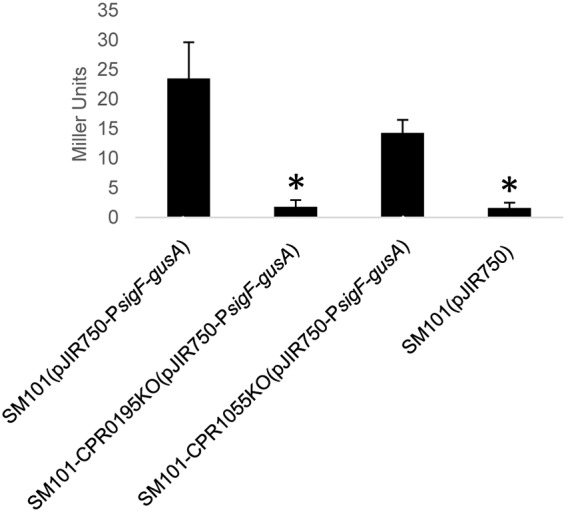
The *cpr0195*-encoded protein affects early steps in sporulation. WT SM101, SM101-CPR0195KO, and SM101-CPR1055KO were each transformed with a pJIR750 shuttle plasmid containing the *gusA* gene under the control of the promoter for the *spoIIA* operon encoding SigF, which is an early sporulation-associated sigma factor necessary for production of SigE, SigG, and SigK (16). Supernatants from overnight MDS cultures of each strain were harvested, the substrate 4-nitrophenyl-β-d-glucuronide was added for 30 min at 37°C, and absorbance was read at 405 nm, and Miller unit values were calculated and plotted. Shown are the mean results from three repetitions of this experiment. Error bars show standard errors. The asterisks (*) indicate *P* values of <0.05 relative to SM101(pJIR750-P*sigF-gusA*).

The results of these analyses, shown in Fig. 5, revealed that, unlike wild-type SM101, the SM101-CPR0195KO mutant demonstrated almost no GusA activity driven by the *spoIIA* promoter of the reporter plasmid. That result indicated that the CPR0195 protein likely regulates very early sporulation steps upstream of the location of SigF production, e.g., during the phosphorylation and activation of Spo0A. When GusA activity was similarly assessed in SM101-CPR1055KO carrying the reporter plasmid, only a partial, statistically insignificant reduction in GusA activity driven by the *spoIIA* operon promoter was observed, which is consistent with the sporulation data shown in Fig. 5.

### CPR0195 affects Spo0A levels in MDS cultures.

**FIG 6:**
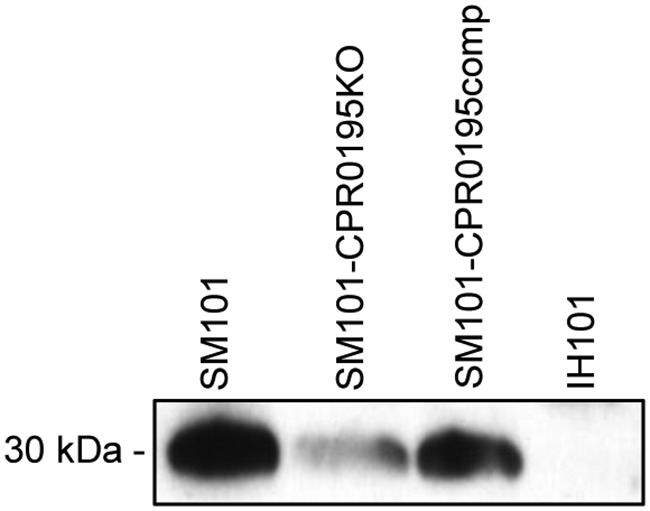
Production of CPR0195 affects Spo0A levels in MDS cultures. Three-hour MDS cultures of the indicated strains were harvested. The OD_600_ was then determined, and the cultures were adjusted to equal OD_600_ levels. Cells were then sonicated to release cellular contents, and the resulting sonicate was subjected to SDS-PAGE and Western blotting with antisera specific to Spo0A. Shown is a representative result for three Western blotting repetitions.

In B. subtilis, phosphorylation of Spo0A results in a feedback loop that increases production of Spo0A (28). To evaluate if CPR0195 similarly affects Spo0A levels in SM101, Spo0A Western blotting was performed to compare Spo0A levels in 3-h MDS cultures of wild-type SM101 versus the isogenic SM101-CPR0195KO null mutant and the SM101-CPR0195comp complementing strain. The results shown in Fig. 6 indicate that SM101-CPR0195KO produces less Spo0A than wild-type SM101 and that this phenotype is reversible by complementation to restore CPR0195 production. Isogenic *spo0A* null mutant IH101 (22), kindly provided by Mahfuzur Sarker, was included as a negative control for this Western blot experiment.

### Evidence that CPR0195 is a kinase that can directly phosphorylate Spo0A.

If CPR0195 is a major kinase initiating sporulation and subsequent CPE production, it would be expected that this protein would be able to phosphorylate Spo0A directly. Such activity has been demonstrated for sporulation-initiating orphan kinases of C. acetobutylicum by showing that the purified kinase domains of these kinases can directly phosphorylate purified Spo0A *in vitro* (18). For this nonpathogenic *Clostridium* spp., this process apparently involves autophosphorylation of the orphan kinases.

**FIG 7:**
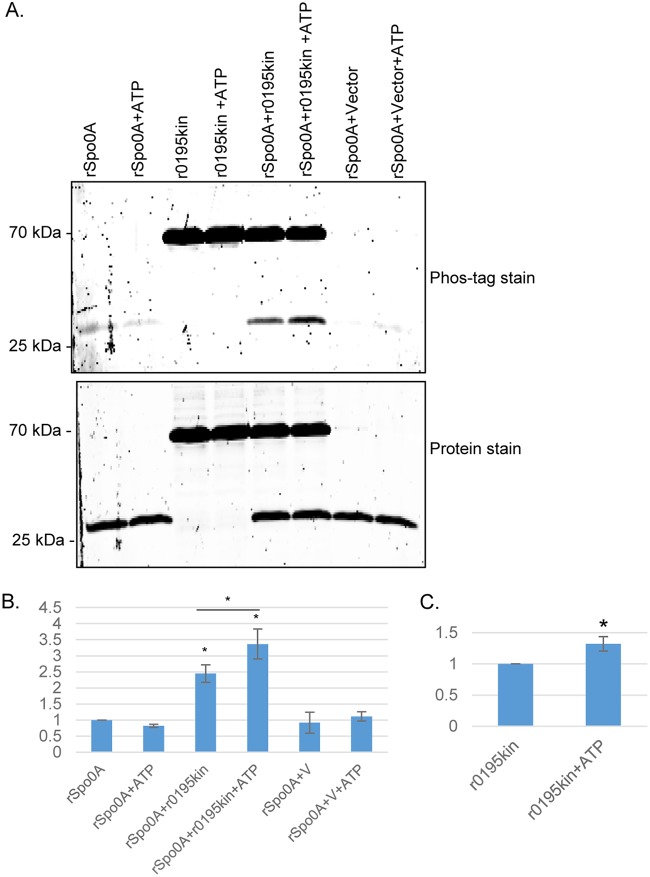
Phosphotransfer from the recombinant CPR0195 kinase domain rCPR0195kin (r0195kin) to recombinant Spo0A (rSpo0A) *in vitro*. Purified r0195kin (0.4 µM) was incubated with rSpo0A (4 µM) in phosphotransfer buffer that did or did not contain (as indicated) ATP at room temperature for 1 h. Those samples were then electrophoresed overnight at 4°C on a 15% acrylamide gel containing SDS. (A) The upper photograph shows a gel stained with Phos-Tag phosphoprotein gel stain; the lower photograph shows the same gel stained with eLuminol protein gel stain to show equivalent loading levels of rSpo0A or r0195kin, as appropriate, in different lanes. (B) Quantitative analysis of rSpo0A phosphoprotein levels with or without r0195kin and in the absence or presence of ATP, as indicated. The band intensities of gels were compared by Image J analysis. *, *P* < 0.05 (in comparison to Spo0A without ATP by ordinary one-way analysis of variance [ANOVA]). #, *P* < 0.05 (in comparison to r0195kin without ATP; Student's *t* test). (C) Quantitative analysis of r0195kin phosphoprotein levels in the absence (left) or presence (right) of ATP. The band intensities of gels were compared by ImageJ analysis. *, *P* < 0.05 (in comparison to r0195kin without ATP; Student's *t* test). All experiments were repeated three times, and mean values are shown in panels B and C. The error bars indicate standard deviations.

CPR0195 is predicted to be a membrane protein, and those are often difficult to clone and analyze in heterologous hosts. In response, previous studies of clostridial orphan histidine kinases (18, 20) used the putative kinase domain of these proteins after production in Escherichia coli. Similarly, the putative recombinant CPR0195 kinase domain (rCPR0195kin) and recombinant C. perfringens Spo0A (rSpo0A) were both expressed in E. coli and purified to apparent homogeneity (Fig. 7A). As noted previously, when B. subtilis Spo0A was expressed in E. coli (29), both rCPR0195kin and rSpo0A were substantially phosphorylated under conditions of expression in E. coli and remained so immediately after purification. It would be difficult to detect phosphorylation using purified rSpo0A that had already been substantially phosphorylated, so the level of rSpo0A phosphorylation was reduced prior to performing the *in vitro* kinase phosphorylation assay. This process involved incubating rSpo0A overnight at 37°C with shrimp phosphatase; the shrimp phosphatase was then removed using an Amicon Ultra-4 centrifugation filter before the rSpo0A preparation was further incubated for 1 to 2 days at 37°C to allow additional spontaneous dephosphorylation.

When the less-phosphorylated rSpo0A was incubated for 60 min at 25°C with rCPR0195kin that had also been partially phosphorylated in E. coli, Phos-Tag staining demonstrated an ∼2.5-fold increase in the phosphorylation of rSpo0A (Fig. 7B). As a negative control to address the possibility that this rSpo0A phosphorylation resulted from a copurifying E. coli kinase contaminant, an equal volume of mock-purified sample from E. coli carrying the vector plasmid alone (i.e., with no *cpr0195* insertion) was similarly analyzed. This sample did not affect rSpo0A phosphorylation. Collectively, these results indicated that rCPR0195kin can directly phosphorylate rSpo0A.

To demonstrate that rCPR0195kin can directly phosphorylate Spo0A using ATP and to assess whether rCPR0195kin autophosphorylates, purified rCPR0195kin was incubated with purified rSpo0A in the presence of ATP. This incubation resulted in a significant increase in Phos-Tag staining of rCPR0195kin (Fig. 7B and C), indicating that this protein can autophosphorylate. Importantly, the copresence of ATP and rCPR0195kin also resulted in a significant increase in the phosphorylation of rSpo0A. As a control, the addition of ATP to rSpo0A alone (no rCPR0195kin) did not increase Phos-Tag staining of rSpo0A. Similarly, the addition of ATP to rSpo0A in the presence of the same volume of a mock-purified sample from E. coli empty vector control did not increase rSpo0A phosphorylation. Together, the results shown in Fig. 7 demonstrate that, using ATP, rCPR0195kin can autophosphorylate and then directly phosphorylate rSpo0A. Protein staining of the same gel (Fig. 7A) confirmed that equal amounts of rSpo0A and rCPR0195kin, as appropriate, had been loaded in the lanes.

## DISCUSSION

Sporulation plays two critical roles in C. perfringens type F enteric infections of humans. First, this process produces spores that contribute to environmental persistence and survival of type F strains in the food environment or the nosocomial environment, thereby enhancing transmission of type F food poisoning and nonfoodborne human GI disease. Second, sporulation is required for production of CPE, the major virulence factor of type F strains. Consequently, how C. perfringens type F strains regulate their sporulation and enterotoxin production has been a subject of several studies, resulting in substantial progress toward unraveling the sporulation pathway of C. perfringens. For example, the role of sporulation-associated sigma factors in sporulation and CPE production has now been delineated (15, 16) and an essential role for Spo0A in sporulation has been established (22). However, the critical initial process by which Spo0A becomes phosphorylated has remained a major gap in understanding C. perfringens sporulation and CPE production despite the potential translational value of blocking this early event to reduce food poisoning and nonfoodborne disease caused by type F strains.

When *Bacillus* spp. initiate their sporulation, the Spo0A master transcriptional regulator becomes phosphorylated via a phosphorelay that is absent from clostridial genomes (12–14). Instead, it is generally accepted that, to initiate sporulation, the clostridia phosphorylate their Spo0A using orphan kinases. Some evidence supports this view. For two nonpathogenic clostridial species, i.e., C. acetobutylicum and C. thermocellum, multiple orphan histidine kinases have been implicated in sporulation (18, 19). For C. acetobutylicum, inactivation of genes encoding any of three different histidine kinases drops sporulation levels moderately, i.e., by 20-fold to 100-fold each (18). It was also shown that two of those kinases can autophosphorylate and then transfer a phosphate group onto Spo0A *in vitro*. Those findings led to the proposal of two sporulation pathways in this *Clostridium* species, one involving a single orphan kinase and the other involving two orphan kinases that act together. Another study (19) showed that, for C. thermocellum, three kinases positively control sporulation and that inactivating the gene encoding one of these histidine kinases essentially abolishes sporulation. However, to our knowledge, it has not yet been shown that any of these C. thermocellum kinases can autophosphorylate or directly transfer a phosphate onto Spo0A.

For the pathogenic clostridia, data from previous studies have provided some support for the involvement of orphan histidine kinases in Spo0A phosphorylation and sporulation (20, 21), although those prior results have not yet been as compelling as those from the nonpathogenic clostridia. For Clostridium botulinum, an orphan kinase was suggested to phosphorylate Spo0A, but the effects of inactivating that kinase gene on sporulation were not assessed; neither was direct phosphorylation of Spo0A by that kinase demonstrated (21). For C. difficile, inactivation of one putative orphan kinase gene reduced sporulation, although only by about 3.5-fold, which indicates that additional kinases are probably also involved in initiating sporulation (20). A different orphan kinase of C. difficile was shown to phosphorylate Spo0A *in vitro*, but the importance of this kinase for sporulation was not assessed using a kinase null mutant (20).

Considering the findings mentioned above, the current report now offers important new insights regarding the involvement of an orphan histidine kinase in initiating sporulation by a pathogenic clostridial species. Specifically, CPR0195 was identified as a critically important kinase for efficient C. perfringens sporulation since inactivation of the *cpr0195* gene reduced sporulation by >1,000-fold. Furthermore, complementation of the *cpr0195* null mutant significantly restored sporulation, indicating that the poor sporulating ability of this mutant was not due to a secondary mutation. It was also shown that mutation of the *cpr0195* gene virtually eliminated CPE production. Thus, if an inhibitor of this membrane-bound kinase could be identified, it might interfere with pathogenesis by reducing intestinal sporulation and CPE production during type F enteric disease.

Beyond establishing an important role for CPR0195 in sporulation and CPE production, this study also demonstrated that the kinase domain of CPR0195 can autophosphorylate and then directly transfer a phosphate onto Spo0A. This information, coupled with the *spoIIA* operon reporter results showing that CPR0195 works very early in sporulation, supports the idea of a role for this kinase in initiating C. perfringens sporulation. Summarizing, by demonstrating (i) that the *cpr0195* null mutant has a profound sporulation defect, (ii) that the CPR0195 kinase domain can autophosphorylate and directly phosphorylate Spo0A, and (iii) that CPR0195 has an early role in sporulation, this report presents the most comprehensive evidence to date that an orphan histidine kinase plays a critical role in initiating sporulation by a pathogenic clostridial species.

This work also attempted to inactivate other putative orphan histidine kinase genes. A mutant was successfully constructed in the gene encoding CPR1055. In contrast to the *cpr0195* mutant, the *cpr1055* null mutant showed only a slight defect in sporulation and CPE production, with neither of those effects reaching statistical significance compared to those seen with the wild-type parent. Those *cpr1055* sporulation and CPE production results provide specificity to the CPR0195 results by demonstrating that inactivating any putative orphan histidine kinase gene of C. perfringens does not necessarily cause a profound defect in CPE production and sporulation. It is worth noting that a review by Talukdar et al. (14) mentioned unpublished data indicating a sporulation defect for a *cpr1055* null mutant. Since that previous finding remains unpublished, it is not possible to assess the scale of the sporulation defect for that prior *cpr1055* mutant or whether that defect reached statistical significance. Neither was it mentioned whether *cpr1055* regulated CPE production. Last, our inability to inactivate the gene encoding CPR1316 might suggest that some of the putative histidine kinase genes are essential for C. perfringens survival. However, it is apparently possible to inactivate the gene for at least some additional putative histidine kinase genes since the review by Talukdar et al. refers to a CPR1728 null mutant, although that result again remains unpublished. So, at this point, it is possible, although unproven, that additional C. perfringens mutants can be constructed for putative orphan kinases and this should be pursued further in the future.

Since the gene encoding CPR0195 appears to be present in most if not all C. perfringens strains, the current findings implicate CPR0195 as a major regulator of sporulation in this bacterium. Interestingly, there does not appear to be a close homologue of the *cpr0195* gene in other clostridial spp., supporting the idea of differences in sporulation initiation among these bacteria. However, despite the profound impact of CPR0195 on C. perfringens sporulation, there may be at least one additional sporulation pathway in this bacterium that is mediated by another orphan histidine kinase since the *cpr0195* null mutant still exhibits some sporulation, although at a very low frequency. All seven potential orphan kinases are highly conserved among the seven diverse C. perfringens strains mentioned in Results, with the exception that the *cpr1055* and *cpr131*6 genes are absent from type C strain JGS1495, which sporulates poorly in MDS sporulation medium (data not shown). The absence of the *cpr1316* gene from the minimally sporulating JGS1495 strain might suggest a role for this kinase gene in sporulation, but an alternative possibility is that this strain is defective in expressing some other gene important for sporulation. Those possibilities are likely to be definitively distinguished only by future studies involving a *cpr1316* null mutant.

It could be envisioned that a possible alternative (i.e., a non-CPR0195-mediated) sporulation pathway would become more important under certain environmental conditions. If validated, the use of two pathways to phosphorylate Spo0A and initiate C. perfringens sporulation would resemble the situation in C. acetobutylicum, where, as mentioned earlier, there appear to be two independent pathways leading to Spo0A phosphorylation, i.e., one pathway mediated by a single orphan histidine kinase and the second by two different orphan histidine kinases (18). However, there would still remain a major difference between C. perfringens sporulation initiation and C. acetobutylicum sporulation initiation by orphan histidine kinases; i.e., the *cpr0195* null mutation has a much greater impact on sporulation (this study) than any single orphan kinase mutation reported in C. acetobutylicum (18). As possible, construction and characterization of additional C. perfringens orphan kinase mutants would help to illuminate the issue of possible additional pathways to Spo0A phosphorylation and sporulation initiation in this bacterium.

## MATERIALS AND METHODS

### Bacterial strains, media, and growth conditions.

SM101, a transformable derivative of a C. perfringens type F food poisoning isolate (30), was subjected to insertional mutagenesis and used for all sporulation experiments in this study. CPE-negative C. perfringens type A strain 13 (31) was used as a cloning host during the *cpr0195* complementation vector construction. Fluid thioglycolate broth (FTG; Becton, Dickinson), TGY medium (3% tryptic soy broth [Becton, Dickinson], 2% glucose [Fisher Scientific], 1% yeast extract [Becton, Dickinson], 0.1% sodium thioglycolate [Sigma-Aldrich]), and brain heart infusion (BHI) agar (Research Products International) were used for vegetative culture of C. perfringens. For sporulating cultures of C. perfringens, all strains were grown in modified Duncan-Strong medium (MDS) supplemented with caffeine and raffinose (1.5% proteose peptone [Becton, Dickinson], 0.4% yeast extract [Becton, Dickinson], 1% disodium phosphate heptahydrate [Fisher Scientific], 0.1% sodium thioglycolate [Sigma-Aldrich], 1 mM caffeine [Sigma-Aldrich], 0.4% raffinose [Sigma-Aldrich]).

E. coli strains DH5α and NiCo21(DE3) (New England Biolabs) were used as a cloning host and for recombinant protein expression and purification. E. coli was grown in Luria-Bertani broth (LB; 1% tryptone [Becton, Dickinson], 0.5% yeast extract [Becton, Dickinson], 1% NaCl [Fisher Scientific]) or on LB agar (LB containing 1.5% agar).

### Plasmid construction.

All primers used in the study, and the expected sizes of the PCR products obtained using those primers, are listed in Table 1. Plasmids pJIR750_0195i and pJIR750_1055i were constructed to prepare *cpr0195* and *cpr1055* null mutants using the *Clostridium*-modified TargeTron gene knockout system (32). The intron targeting the *cpr0195* gene was prepared using primers 0195-706/707s-IBS, 0195-706/707s-EBS1d, and 0195-706/707s-EBS2 (Table 1). The resultant 350-bp PCR product was ligated into pJIR750ai between the HindIII and BsrGI restriction sites to create pJIR750_0195i. The intron targeting the *cpr1055* gene was prepared using primers 1055-433/434s-IBS, 1055-433/434s-EBS1d, and 1055-433/434s-EBS2 (Table 1). The resultant 350-bp PCR product was ligated into pJIR750ai between the HindIII and BsrGI restriction sites to create pJIR750_1055i. The intron-carrying plasmids were transformed into E. coli DH5α, and transformants were then plated onto LB agar containing 50 μg/ml of chloramphenicol.

**TABLE 1:** Primers used in this study

Primer name	Primer sequence	Purpose	PCR product size (bp)
0195-706/707s-IBS	AAAAAAGCTTATAATTATCCTTAGGATTCGTGAAGGTGCGCCCAGATAGGGTG	pJIR750_0195i construction	350
0195-706/707s-EBS1d	CAGATTGTACAAATGTGGTGATAACAGATAAGTCGTGAAGTATAACTTACCTTT CTTTGT	pJIR750_0195i construction	
0195-706/707s-EBS2	TGAACGCAAGTTTCTAATTTCGATTAATCCTCGATAGAGGAAAGTGTCT	pJIR750_0195i construction	

0195KOF	CATAAAACAATTTCATCAGTAGCAG	Screen for intron insertion in *cpr_0195*	350
0195KOR	AAAACTCCACTTATAGCCAATAAAA	Screen for intron insertion in *cpr_0195*	

1055-433/434s-IBS	AAAAAAGCTTATAATTATCCTTAAATAACG-ATATAGTGCGCCCAGATAGGGTG	pJIR750_1055i construction	350
1055-433/434s-EBS1d	CAGATTGTACAAATGTGGTGATAACAGATAAGTCGATATATTTAACTTACCTTT CTTTGT	pJIR750_1055i construction	
1055-433/434s-EBS2	TGAACGCAAGTTTCTAATTTCGGTTTTATTCCGATAGAGGAAAGTGTCT	pJIR750_1055i construction	

1055Fscreen	CTTTTAATCATATTGACAGAAAA	Screen for intron insertion in *cpr_1055*	319
1055Rscreen	TTAGGATGGATTCTATCTAATTTTAC	Screen for intron insertion in *cpr_1055*	

0195compF	CATCATGGATCCGATACATTTAATAATTCTGCAAATTCTTTAGAC	pJIR750_0195comp construction	3,474
0195compR	CATCATGGATCCGAGACAAAAGTAAGTTTTGATATACC	pJIR750_0195comp construction	

PJIR750Screen	CTGCAGGTCGACTCTAGA	Screen for pJIR750_ 0195comp in Strain 13	

0195RT-F	CAAAGAAATCTTTAGATGATTGCGA	RT-PCR analysis of *cpr_0195*	329
0195RT-R	GATAGAAAACTTGGAGTTCATTTTGTAG	RT-PCR analysis of *cpr_0195*	

1055RT-F	GCAAAGGTTTAAATGAGAGTTTTAG	RT-PCR analysis of *cpr_1055*	282
1055RT-R	TATAGAATATATTCTCTGCTATTCCAC	RT-PCR analysis of *cpr_1055*	

PolCF	ACTTCCCTGCAAGCCTCTTCTCCT	RT-PCR analysis of *polC*	393
PolCR	TGGTTCAGCTTGTGAAGCAGGGC	RT-PCR analysis of *polC*	

rSpo0AHisF	CCCCTCTAGAAATAATTTTGTTTAACTTTAAGAAGGAGATATACCATGAAGGAAT CAAAAATATCTGTAT	Cloning of *spo0A* gene for recombinant expression	828
rSpo0AHisR	GTGCTCGAGGCTAACTTTATTCTTTAGTCTTAATTTATC	Cloning of *spo0A* gene for recombinant expression	

r0195HisF	CCCCTCTAGAAATAATTTTGTTTAACTTTAAGAAGGAGATATACCATGGAAATTA CGACTAAAAACAAAAAAAG	Cloning of *cpr_0195* gene for recombinant expression	1,543
r0195HisR	GTGCTCGAGATGTACTTCATAAATATCAGAAAGCTC	Cloning of *cpr_0195* gene for recombinant expression	

The *cpr0195* complementation vector, which was named pJIR750_0195comp, was constructed as follows. A region of the SM101 genome (23) spanning the 800 bp upstream of the *cpr0195* ORF, the *cpr0195* ORF itself, and 300 bp downstream of the *cpr0195* ORF was PCR amplified using primers 0195compF and 0195compR (Table 1). The resultant PCR product was then ligated into pJIR750 between the SalI and PstI restriction sites before electroporation of the *cpr0195*-carrying plasmid into C. perfringens strain 13 and selection of transformants on BHI agar containing 15 μg/ml of chloramphenicol.

Sequences encoding Spo0A or the predicted kinase domain of CPR0195 were PCR amplified using primer pairs rSpo0AHisF/rSpo0AHisR and r0195HisF/r0195HisR. Those PCR products were then ligated into pET28(a)^+^ to create pET28(a)^+^-*spo0A* and pET28(a)^+^-0195. Those plasmids were transformed into E. coli NiCo21(DE3), and the transformants were then plated on LB agar containing 100 μg/ml of ampicillin.

### Construction of *cpr0195* or *cpr1055* null mutants and a *cpr019*5 complementation strain.

The *cpr0195* and *cpr1055* genes were insertionally inactivated in C. perfringens strain SM101 using a targeted group II intron (32). Plasmid pJIR750_0195i was electroporated into wild-type SM101, and the resultant transformants were plated onto BHI agar containing 15 μg/ml of chloramphenicol. The identity of mutant colonies containing an intron insertion between base pairs 706 and 707 of the *cpr0195* open reading frame (ORF) was confirmed by PCR using primers 0195Fscreen and 0195Rscreen (Table 1; see also Results). The intron-containing plasmid was cured from one mutant, which was named SM101-CPR0195KO. The mutant was then assessed for a single intron insertion by Southern blotting, for elimination of gene expression by RT-PCR (see below), and for growth characteristics by growth curve analysis.

Similarly, pJIR750_1055i was electroporated into wild-type SM101 and the resultant transformants were then plated onto BHI agar containing 15 μg/ml of chloramphenicol. Mutant colonies containing an intron insertion in the *cpr1055* ORF between base pairs 433 and 434 were confirmed by PCR using primers 1055Fscreen and 1055Rscreen (Table 1; see also Results). After curing of the plasmid from one mutant (named SM101-CPR1055KO), that mutant was then analyzed exactly as described above for SM101-CPR0195KO.

The SM101-CPR0195KO mutant was electroporated with plasmid pJIR750_0195comp and then plated onto BHI containing 15 μg/ml of chloramphenicol to create the complementing strain SM101-CPR0195comp. SM101-CPR0195comp was characterized by PCR for the presence of the intact *cpr0195* gene, by RT-PCR for the confirmation of *cpr0195* expression, and by growth curve analysis to assess growth characteristics.

### DNA isolation, PCR, and Southern blot analysis.

Genomic DNA was isolated from all strains using a MasterPure Gram-Positive DNA purification kit (Epicentre), as previously described (25). Plasmid DNA was isolated from E. coli using a QIAprep Spin Miniprep Kit (Qiagen). Plasmid DNA was isolated from C. perfringens using methods described previously (33) before electroporation into SM101-CPR0195KO.

The PCR for preparing the complementing strain used LongAmp *Taq* polymerase (New England Biolabs) and the following parameters: (i) 95°C for 4 min; (ii) 35 cycles of 95°C for 30 s, 50°C for 30 s, and 65°C for 2 min; and (iii) a final extension of 65°C for 10 min. PCRs for screening mutants/transformants were performed using 2× MasterMix *Taq* polymerase (New England Biolabs) and the following parameters: (i) 94°C for 4 min; (ii) 35 cycles of 94°C for 30 s, 50°C for 30 s, and 68°C for 2 min; and (iii) a final extension of 68°C for 10 min.

To confirm the insertion of only a single intron into SM101-CPR0195KO or SM101-CPR1055KO, Southern blot analysis was performed as previously described (16). Briefly, purified genomic DNA from each strain was digested with EcoRI (New England Biolabs), electrophoresed on an agarose gel, and transferred to a positively charged nylon membrane (Roche) using alkali transfer. An intron-specific DIG-labeled probe was then incubated with the blot, and the hybridized probe was detected with CPSD substrate (Roche).

### RNA isolation, RT-PCR, and reverse transcription-quantitative PCR (qRT-PCR) analyses.

RNA was isolated as previously described (34). Briefly, for expression screening, wild-type SM101 was grown in MDS medium for 2 or 4 h or TGY media for 2, 4, or 8 h at 37°C. For confirming insertional mutagenesis by the TargeTron system, wild-type SM101, SM101-CPR0195KO, SM101-CPR0195comp, and SM101-CPR1055KO strains were grown in MDS medium for 2 h. Cultures were then pelleted at the indicated times, and RNA was extracted using saturated phenol. After extraction with phenol, the RNA-containing aqueous phase was twice extracted using TRIzol and chloroform. Following treatment with DNase to remove any residual DNA, RNA was quantified by determining the absorbance at 260 nm. Next, 1 µl of purified RNA was used in a one-step RT-PCR containing 2× *Taq* Master Mix (New England Biolabs), MilliQ water, and primers specific for the *cpr0195* gene, *cpr1055* gene, or *polC* gene (Table 1). Duplicate reaction mixtures were set up, one containing avian myeloblastosis virus (AMV) reverse transcriptase (4 U; Promega) and the other without AMV reverse transcriptase as a control. Reaction mixtures were incubated for 45 min at 37°C to allow cDNA synthesis, before PCR cycling as follows: (i) 95°C for 4 min; (ii) 35 cycles of 94°C for 30 s, 50°C for 30 s, and 68°C for 30 s; and (iii) a final extension of 68°C for 10 min.

### Measurement of C. perfringens growth.

The growth characteristics of wild-type SM101, SM101-CPR0195KO, SM101-CPR0195comp, and SM101-CPR1055KO were determined as previously described (27). Briefly, 0.2 ml of an overnight FTG culture of each strain was inoculated into 10 ml of MDS or TGY medium. The optical density at 600 nm (OD_600_) of each culture was then determined at 0, 2, 4, 6, or 8 h using a Bio-Rad SmartSpec spectrophotometer.

### Enumeration of heat-resistant spores.

To enumerate the heat-resistant spores formed, 0.4-ml aliquots from overnight FTG cultures of wild-type SM101, SM101-CPR0195KO, SM101-CPR0195comp, or SM101-CPR1055KO were inoculated into 10 ml of MDS medium. After overnight growth at 37°C, each culture was heated at 70°C for 20 min to kill vegetative cells and to stimulate spore germination. The heat-shocked cultures were then diluted with sterile water and plated on BHI plates. After overnight incubation in an anaerobe jar at 37°C, the resultant colonies on each BHI agar plate were counted.

### Evaluation of CPE production by Western blotting.

A 0.4-ml aliquot from an overnight FTG culture of wild-type SM101, SM101-CPR0195KO, SM101-CPR0195comp, or SM101-CPR1055KO was inoculated into 10 ml of MDS medium. After overnight growth at 37°C, equal volumes of culture (containing cells and supernatant) were lysed in 6× Laemmli SDS-PAGE reducing sample buffer. Equal amounts of the resulting lysates were loaded onto 10% polyacrylamide gels and separated by SDS-PAGE. Following transfer to nitrocellulose membranes, the membranes were blocked and probed using rabbit anti-CPE polyclonal antisera (34) and blots were developed as previously described (16).

### GusA reporter assays.

pJIR750-P*sigF* GusA reporter plasmid (27) was electroporated into wild-type SM101, SM101-CPR0195KO, or SM101-CPR1055KO. The empty pJIR750 plasmid was also electroporated into SM101 as a negative control. Transformants were then plated onto BHI agar containing 15 μg/ml of chloramphenicol before overnight growth in an anaerobe jar at 37°C. To measure GusA activity in each transformant culture, supernatants from overnight MDS cultures of each strain containing pJIR750-P*sigF* were collected. After collection of the culture supernatants, 50 μl of 6 mM 4-nitrophenyl-β-d-glucuronide (in phosphate-buffered saline [PBS] buffer) was added to 250 μl of the supernatant of an overnight culture and incubated for 30 min at 37°C. At that time, the absorbance at 405 nm was read and the GusA activities calculated, and the results are presented as Miller specific activity units (27).

### Determination of Spo0A levels in C. perfringens MDS cultures.

Three-hour MDS cultures of SM101, SM101-CPR0195KO, SM101-CPR0195comp, or IH101 (a previously described *spo0A* deletion mutant [22]) were collected by centrifugation. Following resuspension in PBS, the OD at 600 nm was determined and culture densities were adjusted to equal OD_600_ levels. Equal volumes of the OD_600_-adjusted cultures were then removed and lysed by sonication, and 6× Laemmli SDS-PAGE reducing sample buffer was added to the lysates. Following boiling for 5 min at 95°C, lysates were loaded onto a 10% polyacrylamide gel and separated by SDS-PAGE. The separated lysate proteins were transferred onto nitrocellulose, and the resultant blots were probed with polyclonal rabbit anti-Spo0A (35) that was kindly provided by Masaya Fujita. Bound anti-Spo0A antibody was detected using a horseradish peroxidase (HRP)-conjugated goat anti-rabbit secondary antibody, and Spo0A was detected using GE Healthcare Western blotting substrate.

### Expression and purification of recombinant Spo0A (rSpo0A) and the recombinant CPR0195 kinase domain (rCPR0195kin).

rSpo0A or rCPR0195kin was expressed in E. coli NiCo21(DE3) cells using the pET28a^(+)^ (Novagen) expression system. Brieﬂy, primers (Table 1) were designed to amplify the *spo0A* open reading frame (ORF) or the kinase domain of the CPR0195 ORF, as predicted by the PSORTb program. After PCR amplification using DNA from C. perfringens strain SM101, products were digested with XbaI and XhoI (New England BioLabs) and ligated into pET28a^(+)^ encoding an C-terminal His_6_ tag, and the ligation mixture was transformed into E. coli NiCo21(DE3) cells to sharply reduce the level of background copurifying contaminants. Successful constructs were conﬁrmed by DNA sequencing at the Genomics Research Core at the University of Pittsburgh.

For purification of these recombinant protein species, E. coli NiCo21(DE3) transformants producing rSpo0A or rCPR0195kin or carrying the pET28a^(+)^ empty vector (as a negative control) were grown overnight at 37°C with aeration in 20 ml of LB broth supplemented with 100 µg/ml of kanamycin (Km). This starter culture was then used to inoculate 1 liter of LB broth supplemented with 100 µg/ml of Km. After 6 h of incubation at 25°C, isopropyl-β-d-thiogalactopyranoside (IPTG) was added to the cultures at a ﬁnal concentration of 1 mM and incubation was continued overnight at room temperature. After this ﬁnal incubation, cells were harvested by centrifugation and pelleted cells were resuspended in HTBB buffer (20 mM NaH_2_PO_4_, 500 mM NaCl, 40 mM imidazole, pH 7.4) containing proteinase inhibitor. Those suspensions were then lysed on ice by using a Qsonica sonicator (5-min total run time with 40% maximum output) and centrifuged for 20 min at 15,500 rpm. Lysate supernatants were added to 1 ml of nickel-nitrilotriacetic acid (Ni-NTA) resin (Clontech) and incubated for 30 min at 4°C. Following this incubation, the resin was loaded onto a gravity column and washed with 20 ml of HTBB buffer. Bound protein was eluted from the column using 3 ml of HTBB buffer containing 500 mM imidazole. The collected eluent was then mixed with 1 ml of chitin beads (New England Biolabs), which were prewashed with buffer containing 50 mM sodium phosphate and 500 mM sodium chloride (pH 7.4), and the mixture was gently shaken overnight at 4°C. The mixture was then added to a column, and the flowthrough was collected and desalted into 20 mM Tris-HCl buffer using PD-10 desalting columns (GE Healthcare). The final protein concentration of the purified protein was determined using a protein bicinchoninic acid (BCA) assay (Thermo Fisher). After similar processing of lysates from an equivalent volume (as used for purifying rCPR0195kin) of an empty vector-carrying E. coli control culture was performed, the same volume of this mock-purified preparation was used in all studies as a control for background E. coli activities.

### Phosphorylation assays.

Because Phos-Tag staining indicated that rSpo0A was already strongly phosphorylated when it was produced in E. coli, which would make it difficult to detect its phosphorylation by rCPR0195kin, the level of rSpo0A phosphorylation was reduced prior to its use in an rCPR0195kin phosphorylation assay. This was accomplished by concentrating purified rSpo0A, with buffer exchange to Smart Cut buffer (New England Biolabs), from 3.5 ml to 0.9 ml using an Amicon Ultra-4 ultrafiltration device (10-kDa cutoff). A 100-μl aliquot of shrimp phosphatase (New England Biolabs) was added to that sample, which was then incubated overnight at 37°C. The sample was then subjected to ultrafiltration using an Amicon Ultra-4 ultrafiltration device (50-kDa cutoff) and the flowthrough (containing rSpo0A) was collected. That flowthrough sample was divided into aliquots and stored briefly at −80°C before use.

Protein phosphorylation assay was carried out using kinase buffer (25 mM Tris-HCl, 5 mM β-glycerophosphate, 0.1 mM Na_3_VO_4_, 10 mM MgCl_2_, freshly prepared 2 mM dithiothreitol [Fisher Scientific], pH 7.4) with or without freshly prepared 1 mM ATP disodium salt hydrate (Sigma-Aldrich). Purified rCPR0195kin (0.4 µM), alone or in combination with the less extensively phosphorylated rSpo0A (4 µM), prepared as described above, was incubated at 25°C for 1 h. A 20-µl aliquot of those samples was added to 5 µl 5× SDS loading buffer and then electrophoresed overnight at 4°C on a SDS-containing 15% polyacrylamide gel (at 40 V). The gel was then stained using Phos-Tag phosphoprotein gel stain (GeneCopoeia) according to the manufacturer’s protocol and was imaged using a Typhoon 9400 variable mode imager (Amersham Biosciences), a fluorescence emission filter at 560 nm (long pass), and an excitation filter with a 532-nm laser. The same gel was then stained and imaged using eLuminol protein gel stain (GeneCopoeia) to detect total protein.

### Photomicroscopy.

Wild-type SM101, the SM101-CPR0195KO mutant, or the SM101-CPR0195comp strain was grown overnight at 37°C in MDS medium. Aliquots of those cultures were then viewed at ×1,000 by phase-contrast microscopy using a Zeiss AxioVert 200 inverted microscope. The program AxioVision (release 4.8.2) was used to capture images of C. perfringens at that magnification.
